# On glyphosate–kaolinite surface interactions. A molecular dynamic study

**DOI:** 10.1111/ejss.12971

**Published:** 2020-05-06

**Authors:** Edgar Galicia‐Andrés, Daniel Tunega, Martin H. Gerzabek, Chris Oostenbrink

**Affiliations:** ^1^ Institute of Molecular Modeling and Simulation University of Natural Resources and Life Sciences Vienna Austria; ^2^ Institute of Soil Research University of Natural Resources and Life Sciences Vienna Austria; ^3^ School of Pharmaceutical Science and Technology Tianjin University Tianjin China

**Keywords:** adsorption free energy, clay mineral interface, herbicide, molecular modeling, polarization

## Abstract

Glyphosate is an important and widely used herbicide, its environmental behaviour being of scientific and public interest. Computational models of clay minerals and their interactions with small organic molecules are valuable in studying adsorption processes at an atomistic resolution. We analysed the adsorption process of glyphosate on kaolinite, a clay mineral with a high abundance in several types of soils (e.g., of subtropical or tropical origin), in terms of the adsorption strength. The molecular interactions are characterized by monitoring the occurrence of hydrogen bonds, the orientation of the molecular dipole relative to the interface and the interaction energy. Two different ionic forms of glyphosate were considered: neutral and anionic (−1). It was shown that the main mechanism of the binding of both glyphosate forms to the aluminol surface of kaolinite is through multiple hydrogen bonds. The standard free energy of adsorption of neutral glyphosate from water solution to the basal octahedral surface of kaolinite was computed at −5 kJ mol^−1^, whereas for the anionic form this quantity amounted to −14 kJ mol^−1^. Our finding showed that kaolinite has an important contribution to overall adsorption capacity of soils for glyphosate, specifically in its anionic form.

**Highlights:**

- The adsorption free energy of glyphosate on a kaolinite surface is quantified
- Interactions are computed by quantum mechanics and by classical force field
- Molecular interactions are characterized in terms of hydrogen bonds and orientations
- The effect of polarization of the medium on the calculations is analysed

## 1 INTRODUCTION

Glyphosate (*N*‐(phosphonomethyl)‐glycine) is a non‐selective herbicide for weed elimination, which has been widely used in recent history (Duke, 2018; Duke & Powles, 2008). Specifically, no‐till agricultural management systems often rely on glyphosate for weed control. In some countries, it contributes more than 50% of all herbicide applications (Okada, Costa, & Bedmar, 2019). It seems that glyphosate remains in soil for longer time periods, posing potential risks to important soil functions (Gerzabek et al., 2019). During the last few years, concerns have increased worldwide about potential direct and/or indirect health effects of the large‐scale use of glyphosate. A long‐term application of glyphosate may compromise soil and soil‐dependent life forms due to its mobility, the toxicity of its breakdown product aminomethyl phosphonic acid (AMPA) appearing in soils and water sources, its capability to keep residual activity towards some plant species, the toxicity to macro‐ and microorganisms, direct effects on microbial compositions, and potential indirect effects on plants, and animal and human health (Van Bruggen et al., 2018).

Glyphosate has a highly polar character due to its three polar functional groups (amino, carboxylic and phosphonic), with different ionic states depending on pH (pKa_1_ < 2, pKa_2_ = 2.6, pKa_3_ = 5.6 and pKa_4_ = 10.6) (Sprankle, Meggitt, & Penner, 1975). Because of its chemical properties, it is strongly bound to soil constituents and highly soluble in water. Glyphosate may be adsorbed on soils through covalent and/or electrostatic interactions but also through relatively weak interactions such as hydrogen bonds (H‐bonds) on soil humic materials (Piccolo & Celano, 1994; Piccolo, Celano, & Conte, 1996). The strong binding to mineral surfaces is commonly taken as an important factor for the deactivation of glyphosate in soil, which leads to a reduction of its mobility and herbicide efficiency. Consequently, understanding the nature and extent of the interaction of glyphosate with soil mineral surfaces is a key point in investigating the impact and fate of glyphosate in the environment. Kaolinite (Al_2_Si_2_O_5_(OH)_4_) is one of the most common soil minerals belonging to the clay family that was found in several types of soils, for example, of a tropical or subtropical nature.

Adsorption of glyphosate to soil minerals was studied in several experimental works. McConnell & Hossner (1985) demonstrated glyphosate adsorption to kaolinite as a function of pH, and for smectite clays with changes in saturation cations. Glass (1987) reported the adsorption of glyphosate in different soils and clay minerals (montmorillonite, illite and kaolinite) with polyvalent cations, concluding that glyphosate adsorbs within the interlayer spaces of the clay minerals and hypothesizing that glyphosate is complexed by cations released from the clays via a cation‐exchange reaction with solution protons.

Molecular modelling studies on adsorption of glyphosate also exist. Recently, Ahmed, Leinweber, and Kühn (2018) reported an in‐depth study on the nature of glyphosate adsorption to soil mineral goethite (iron oxyhydroxide) by using periodic density functional theory (DFT)‐based molecular dynamics (MD) simulations with three different goethite surface planes ((100), (010) and (001)) in the presence of water. Ahmed et al. observed that the phosphonate functional group is responsible for the complexation process with the goethite surface, in agreement with experimental results (Barja & dos Santos Afonso, 2005). Two different inner‐sphere complexes were observed with different preferential adsorption. H‐bonds played a crucial role in the adsorption and complexation processes, with protons being transferred from glyphosate but still in contact with the glyphosate due to the H‐bond formation with the goethite surface. In addition, Ahmed et al. reported replacement of water molecules with glyphosate at the goethite surface.

In this paper we focused on kaolinite, as one of the most typical soil minerals in subtropical and tropical soils, which often are subject to no‐till management and significant herbicide applications. In a recent study on soil samples from the Galápagos Islands it was shown that glyphosate was massively applied in the past and volcanic soils from these islands still contain a high amount of this herbicide (Gerzabek et al., 2019). Further, it was evidenced that some of these soils were heavily weathered (Zehetner et al., 2020), being rich in kaolinite.

In our previous paper (Galicia‐Andrés, Petrov, Gerzabek, Oostenbrink, & Tunega, 2019), we reported how polarization enhances the adsorption mechanism of water molecules, ions and glyphosate molecules on kaolinite by using classical (force‐field (FF)) MD simulations. Adsorption free energies for the aluminol (001) surface of kaolinite were obtained for glyphosate in neutral C_3_H_8_NO_5_P and one ionic state, C_3_H_7_NO_5_P^−^ (corresponding to pH of ~3 and ~ 6, respectively). In this paper we investigate the molecular basis of the interactions that take place between glyphosate and kaolinite and the role of H‐bond formation as the precursor stage to the subsequent complex formation. FF‐MD simulations are complemented by first principle calculations using a DFT approach.

## 2 MATERIAL AND METHODS

### 2.1 Molecular mechanics setup

Kaolinite, as a 1:1 dioctahedral clay mineral, has an asymmetric composition of layers that consists of tetrahedral (SiO) and octahedral (AlOH) sheets connected through apical oxygen atoms. Thus, the kaolinite layer has two basal surfaces: aluminol, terminated with surface hydroxyl groups, and siloxane, terminated by surface basal oxygen atoms. The layers in the kaolinite structure stay together through hydrogen bonds between the surface hydroxyl groups and the plane of the basal oxygen atoms (Bish, 1993; Neder et al., 1999). It was shown that the aluminol surface is significantly more attractive for polar species (Hu & Michaelides, 2008; Tunega, Gerzabek, & Lischka, 2004); therefore, in this work we present the results for glyphosate adsorption for this surface.

**FIGURE 1:**
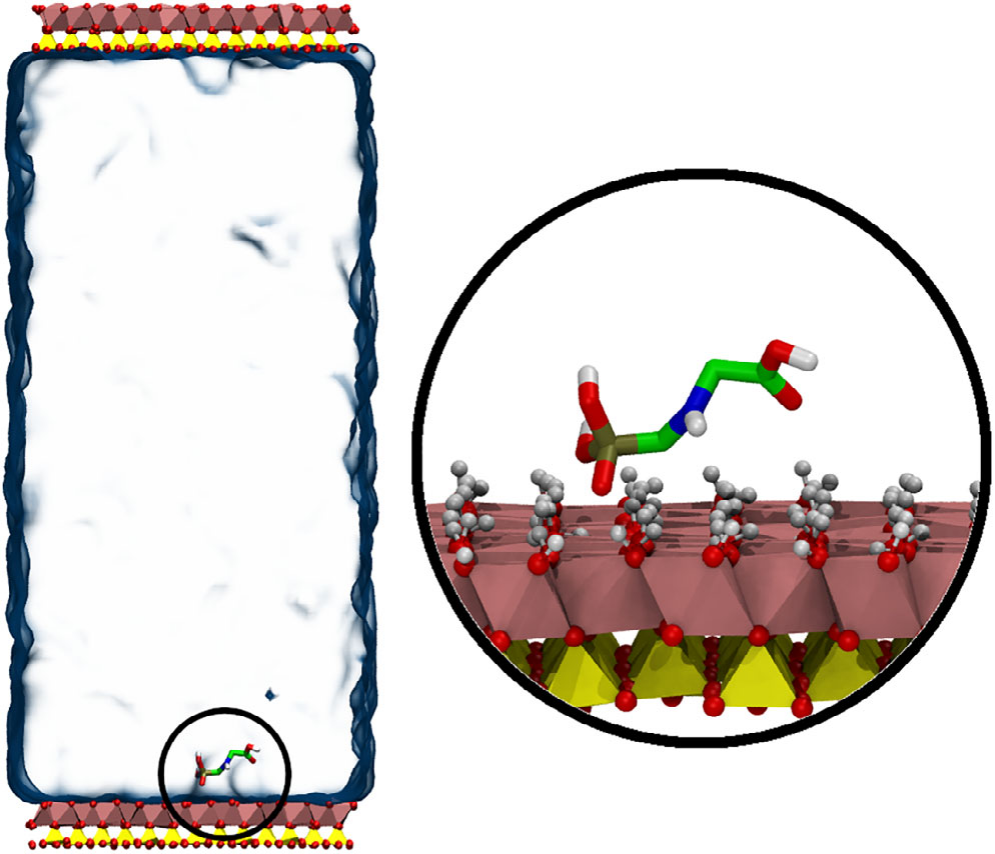
Representation of neutral glyphosate in solution in contact with a kaolinite layer [Color figure can be viewed at wileyonlinelibrary.com]

Heavy atoms of kaolinite were restrained to their ideal positions, *R*_*i*_, by a harmonic potential energy term, *V*(*r*_*i*_) = 1/2 *k* |*r*_*i*_ – *R*_*i*_|^2^ with *k* = 1,000 kJ mol^−1^ nm^−2^, leaving the hydrogen atoms free to move, with the oxygens from the aluminol surface acting in contact with water as H‐bond donor and/or acceptors, respectively (Hu & Michaelides, 2008; Tunega et al., 2004). The simulation box was constructed with two kaolinite layers (each layer containing 28 unit cells (4 × 7 in the *a* and *b* unit cell directions)) separated by a distance of 8 nm, then the simulation box was filled with water molecules in‐between the layers. The glyphosate molecule was placed nearby the aluminol surface, and for charged glyphosates a Na^+^ counterion was placed randomly in the water slab (Figure 1).

The desorption free energy of glyphosate from a kaolinite layer was obtained by umbrella sampling (US) (Torrie & Valleau, 1974). Neutral and ionic states of glyphosate, C_3_H_8_NO_5_P at pKa_2_ = 2.6 and C_3_H_7_NO_5_P^−^ at pKa_3_ = 5.6, respectively, were analysed and compared.

The kaolinite structure was described by the ClayFF (Cygan, Liang, & Kalinichev, 2004) force field and the water by the SPC model (Berendsen, Postma, van Gunsteren, & Hermans, 1981), which is compatible with ClayFF. For glyphosate species and counterions, we used the GROMOS54A8 force field, as in our previous work (Galicia‐Andrés et al., 2019).

MD simulations were performed by the GROMACS package (Abraham et al., 2015) using the leap‐frog algorithm to solve the equations of motion in the canonical ensemble (*NVT*) with a timestep of Δ*t* = 2 fs. The temperature was kept at 300 K using the Berendsen thermostat (Berendsen, Postma, van Gunsteren, DiNola, & Haak, 1984) with a relaxation time constant of τ_*T*_ = 0.1 ps. A cutoff radius of 1.4 nm was used for all non‐bonded interactions with no energy corrections. Long‐range electrostatic interactions were treated using the SPME algorithm (Essmann et al., 1995) with a grid spacing of 0.12 nm and a cubic polynomial. In order to constrain the bond lengths, the LINCS algorithm (Hess, Bekker, Berendsen, & Fraaije, 1997) was used with four matrices in the expansion for the matrix inversion.

The simulation protocol implemented was an energy minimization step with a maximum tolerance of 1,000 kJ mol^−1^ nm^−1^, followed by an equilibration step of 100 ps. The glyphosate centre of mass (COM) was pulled away from the closest mineral layer along a reaction coordinate perpendicular to the layer (along the *z*‐axis) to create the initial configurations for the US method. Up to 44 initial configurations were used with a separation distance of 0.1 nm between each, up to a distance of 4 nm from the aluminol surface. For each configuration, a short equilibration step of 100 ps followed by a production step of 10 ns with a harmonic force constant for the US calculations of 1,000 kJ mol^−1^ was implemented.

Similar to our previous work (Galicia‐Andrés et al., 2019), long‐range interactions were treated by applying two approaches: (a) the conventional Ewald summation technique, periodic in the three dimensions, leading to an induced electric field of confined water, referred as a periodic system, representing a water‐filled nanopore; and (b) a correction applied to the conventional three‐dimensional Ewald summation removing the artificial polarization effect resulting from the periodic repetition, and leading to bulk water interacting with the surface. This model is referred to as the slab system, representing the interactions of a thin water slab with a surface of kaolinite.

### 2.2 DFT setup

DFT calculations were performed with the aim to validate results achieved by the classical FF approach. The DFT calculations were performed on the isolated neutral glyphosate molecule placed on the aluminol surface. The model did not contain any water molecules. The kaolinite was represented by one kaolinite layer containing 2 × 3 unit cells in the *a* and *b* cell directions. Above the surface, a vacuum of about 3.3 nm was imposed to minimize interactions in the *z*‐direction between neighbouring cells. The overall *c* lattice vector was 4 nm. In the first step, the isolated kaolinite layer (without the glyphosate molecule) was optimized together with the lateral *a* and *b* cell dimensions. After adding the glyphosate molecule, the full atomic relaxations were performed, keeping the lattice vectors constant. The DFT calculations were performed by using the Vienna ab initio simulation package (VASP) (Kresse & Furthmüller, 1996; Kresse & Hafner, 1993). The VASP program is developed for electronic structure calculations on periodic structural models based on Kohn‐Sham electron DFT. Exchange‐correlation energy was described by the PBE (Perdew, Burke & Ernzerhof) functional (1996) developed within the frame of the generalized gradient approximation (GGA). The electron–ion interactions were described by atomic pseudopotentials and the projector‐augmented‐wave (PAW) method (Blöchl, 1994; Kresse & Joubert, 1999). The energy cut‐off for the plane‐wave basis set was set up to 400 eV. The geometry relaxation criteria were 10–5 eV for the total electronic energy change and 10–4 eV/Å ~ for the force change. The Brillouin‐zone sampling was restricted to the Γ‐point as we used a large computational cell. Dispersion corrections were added to the PBE functional by using the D3 scheme (Grimme, Antony, Ehrlich, & Krieg, 2010).

## 3 RESULTS

**FIGURE 2:**
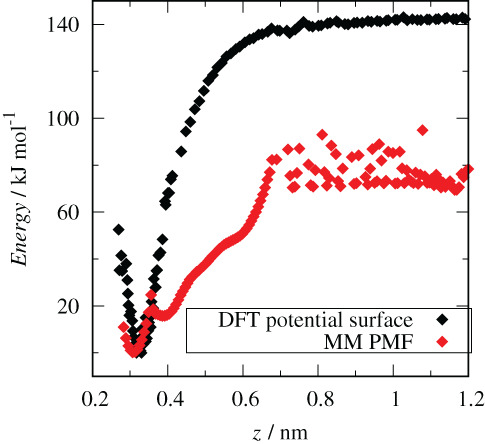
DFT potential energy profile (black diamonds) and PMF (red diamonds) of neutral glyphosate close to the aluminol surface along the *z*‐direction in vacuum. DFT, density functional theory; PMF, potential mean force [Color figure can be viewed at wileyonlinelibrary.com]

In the validation calculations, we determined a vertical interaction potential energy profile from the DFT calculations by placing the glyphosate molecule at a *z*_*i*_ height position and performed relaxations of all atoms in the cell. Then, from the optimized geometry of the system, the potential energy profile was obtained. This procedure was performed by moving of the glyphosate molecule (its COM) from the surface in a perpendicular direction with a step Δ*z* = 0.004 nm until reaching half of the simulation box. On the other hand, we determined the potential mean force (PMF) from the FF‐MD simulations by using umbrella sampling and the weighted histogram analysis method (WHAM) (Kumar, Rosenberg, Bouzida, Swendsen, & Kollman, 1992), using the same structural model as in the DFT calculations. The reaction coordinate in the PMF calculations was defined as a distance in the *z*‐direction between the COM of the glyphosate molecule and the plane of oxygen atoms of the aluminol surface. In order to compare the DFT and PMF energy profiles we set the lowest energy value to zero (Figure 2). We observed that both energy profiles have minima very close to each other, at a distance of ~0.32 nm (DFT) and at ~0.31 nm (PMF).

**FIGURE 3:**
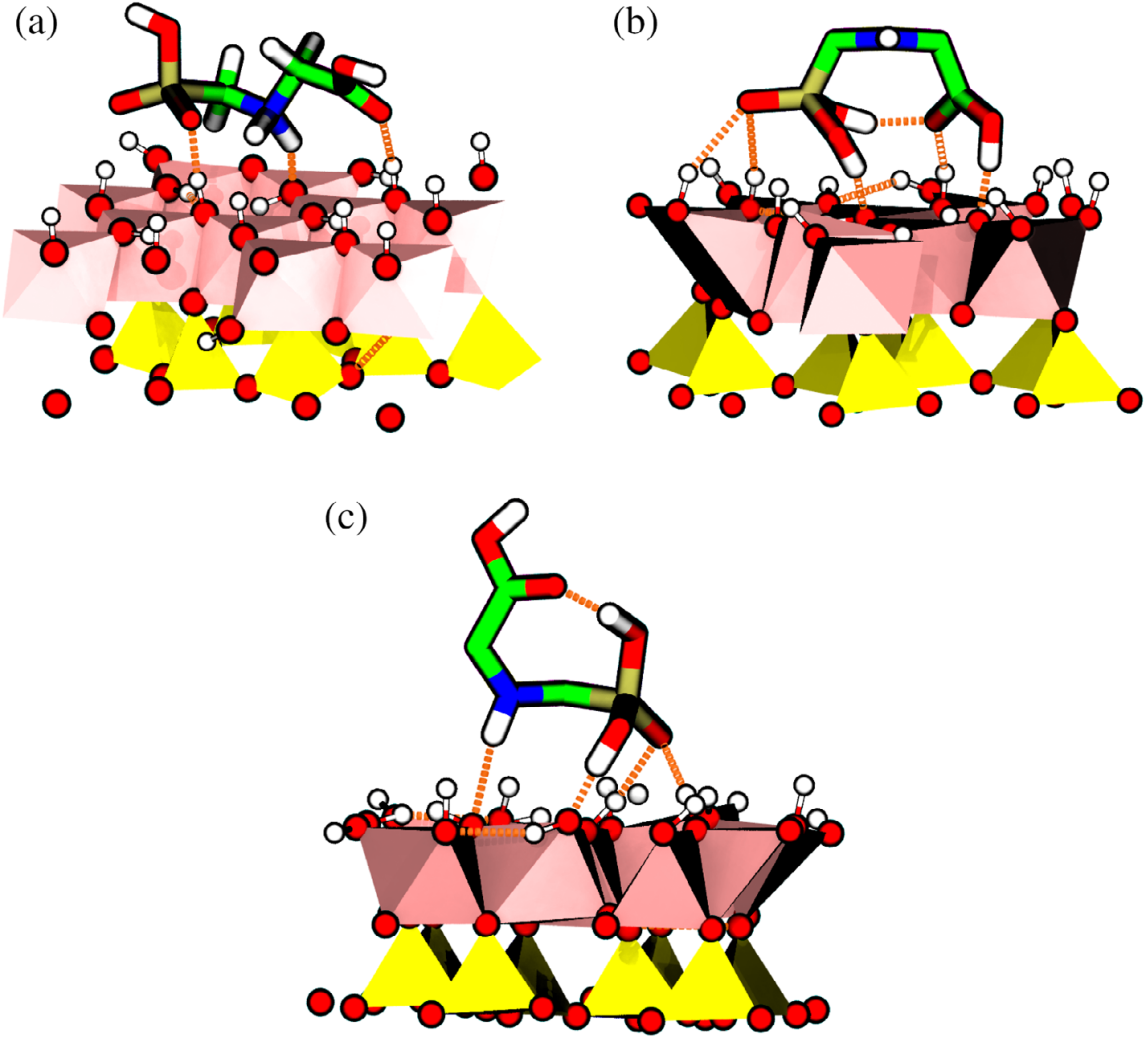
Representation of glyphosate in contact with the aluminol surface. The formed H‐bonds between glyphosate and kaolinite (pink and yellow polyhedra) are represented in the orange colour. Different doses of the glyphosate are displayed: (a) zwitterionic state (DFT); (b) parallel to the surface with both the carboxylate and phosphonate H‐bonding (FF‐MD); (c) phosphonic and amino group H‐bonding (FF‐MD). DFT, density functional theory; FF, force field; MD, molecular dynamics [Color figure can be viewed at wileyonlinelibrary.com]

In both cases, the main molecular axis of the glyphosate molecule is parallel to the aluminol surface, maximizing the formation of H‐bonds of the COOH, NH and PO(OH)_2_ glyphosate groups with the surface hydroxyl groups OH (Figure 3a). In the DFT optimized geometry, formed multiple H‐bonds reached H‐bond lengths of 0.16–0.21 nm. Moreover, DFT calculations showed a proton transfer from PO(OH)_2_ to NH, leading to a zwitterionic form with NH_2_^+^ and PO_2_OH^−^ functionalities. However, once the molecule reached a distance of about 0.39 nm from the surface, the neutral state was recovered in the DFT calculations during the geometry optimization and an intramolecular H‐bond was formed, in agreement with simulations of Ahmed et al. (2018) and experimental results of Sheals, Sjöberg, and Persson (2002), where the formation of intramolecular H‐bonds between phosphonate and amino groups was detected, as well. Contrary to Ahmed et al., the glyphosate molecule was not able to form inner‐sphere complexes, but H‐bonds with the kaolinite surface. O aluminol surface atoms acted as proton donors/acceptors. When the glyphosate distance from the aluminol surface was less than 0.39 nm, glyphosate O atoms acted as proton acceptors, whereas N acted as a proton donor, which seems to promote the intramolecular proton transfer from phosphonate to the amino group (zwitterion state). At distances greater than 0.39 nm glyphosate is still able to H‐bond; however, the neutral glyphosate is the preferred state.

FF‐MD calculations exhibited a surface configuration of the adsorbed glyphosate molecule similar to the DFT results (except for the proton transfer), with glyphosate being able to form a similar hydrogen bonding with the surface hydroxyl groups (Figure 3b). In addition, the PMF curve has a second minimum located at a distance of 0.39 nm. At this distance, the carboxyl group is disconnected from the surface OH groups but H‐bonding between the NH and PO(OH)_2_ groups with surface OH groups remained still feasible (Figure 3c). This conformation was not sampled in the DFT results during the geometry optimization. Both energy profiles show a similar behaviour, with a minimum located at the same position (0.3 nm) and a plateau which appears starting from ~0.7 nm. Differences in the relative energies (60–70 kJ mol^−1^ between both plateaus) can be attributed to the absence of translational and configurational contributions, as well as entropic effects missing in the DFT calculations, which are purely electronic energies not including zero‐point vibrational energy, thermal corrections and standard state correction energies. Adsorption of the glyphosate molecule on the kaolinite surface from gas can be formally considered as an association type of reaction (A + B ⟶ A···B) and missing energetic corrections to the DFT energy can account for values of about 40–50 kJ mol^−1^ (Aquino, Tunega, Haberhauer, Gerzabek, & Lischka, 2002). Looking at the difference between the DFT and PMF plateaus from this perspective we can conclude that the GROMOS force field describes the interactions of the glyphosate molecule with the aluminol surface of the kaolinite layer reasonably.

### 3.1 Adsorption free energy

**FIGURE 4:**
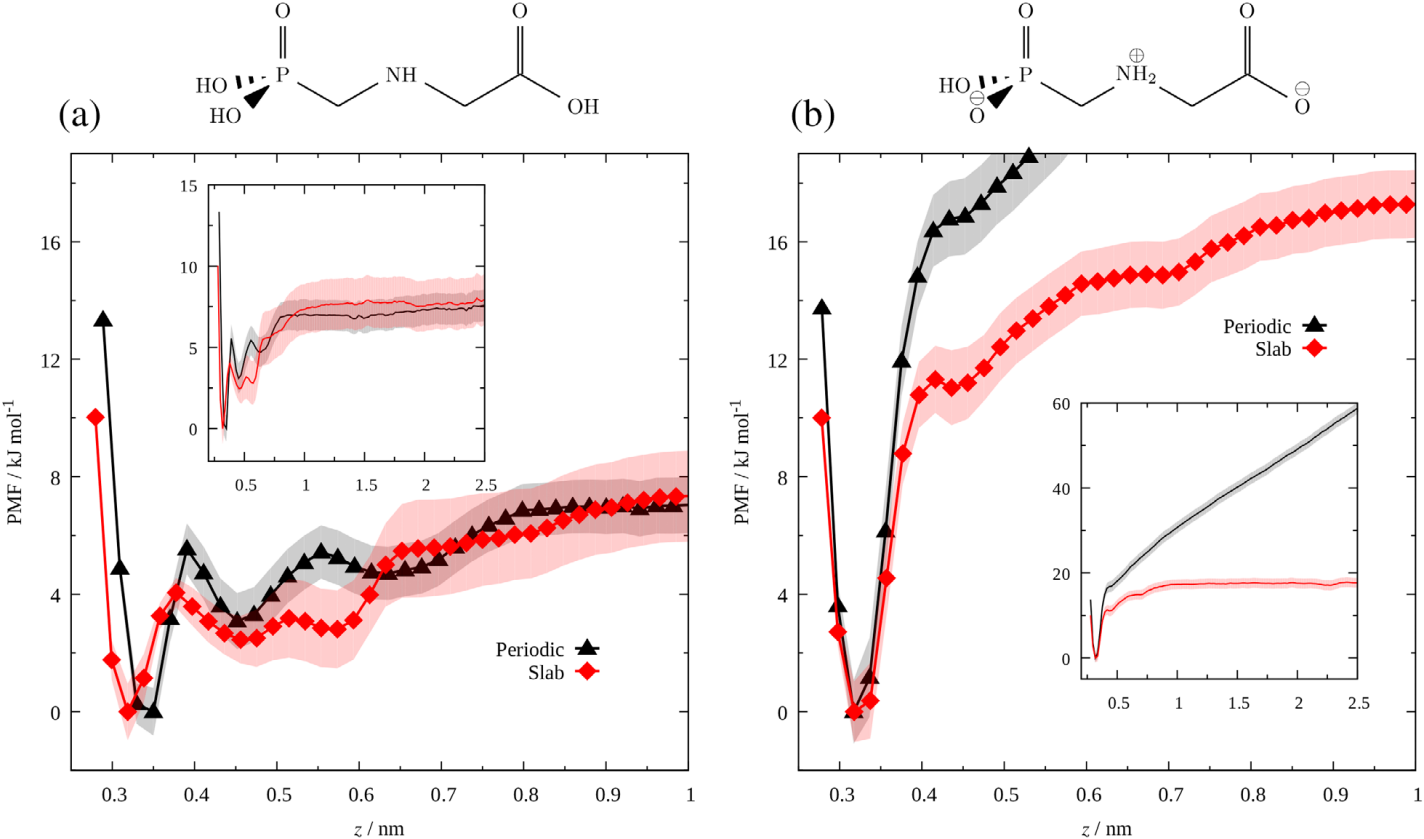
Free energy profile of (a) neutral C_3_H_8_NO_5_P and (b) charged C_3_H_7_NO_5_P^−^ glyphosate species in periodic (black line) and slab (red line) models. For clarity, not all points with error bars are plotted. PMF, potential mean force [Color figure can be viewed at wileyonlinelibrary.com]

To quantify the influence of polarization effects on adsorption processes in explicit water we determined as a first step the PMF curves for both periodic and slab systems. Figure 4 exhibits the PMF as a function of glyphosateʼs COM from the surface for neutral and anionic glyphosate forms. The PMF curve for neutral glyphosate (left panel in Figure 4) has several minima from the surface, particularly at 0.35, 0.45 and 0.62 nm for the periodic model, whereas for the slab model the minima were detected at 0.32, 0.46 and 0.57 nm, respectively. Note that the global minimum in both cases is at a larger distance from the surface than in the case of the isolated glyphosate molecule. This observation can be attributed to the solvation effect of water molecules, which weakens the hydrogen bonding between glyphosate and surface OH groups. Indeed, both PMF curves of the periodic and slab systems have a similar trend, reaching practically the same plateau, starting at a distance of ~0.9–1.0 nm from the surface (Figure 4a), leading to the conclusion that polarization effects have only a minimal impact on the adsorption of the neutral species.

In contrast to the neutral state, the anionic glyphosate form showed two different behaviours at long distances (Figure 4b). For the periodic model, the PMF curve exhibits a continuously increasing trend with increasing distance from the kaolinite surface (black curve in Figure 4b), whereas in the case of the slab model, the PMF levels off at distances beyond 1 nm, similar to the neutral glyphosate (red curve in Figure 4b). Owing to the ionic nature of glyphosate at pH ~ 5 and the effect of polarization, we can conclude that the PMF and the induced electrostatic potential are correlated, which is evidenced by the steepness of curves in Figure 4, enhancing the adsorption mechanism. Additionally, a simulation of the anionic glyphosate with no Na^+^ counterion in the periodic system was performed to quantify if the presence of the counterion interferes with the adsorption, resulting in the same behaviour (data not shown).

It should be noticed that the output of a binding‐pathway free energy method is not a free energy difference (Δ*G*) but a potential of mean force (PMF), which commonly are assumed to be equivalent (Doudou, Burton, & Henchman, 2009). The standard adsorption free energy is calculated from:

(1) $$\Delta G^{0}=\Delta G+\Delta G_{V,}$$

where Δ*G* is obtained from an integration of the PMF and Δ*G*_V_ is a volume correction, relating the unbound state to a standard concentration (1 M). A derivation of the standard adsorption free energy is given in the Supplementary Material. Table 1 shows the final standard adsorption free energies calculated for the periodic and slab systems of the neutral glyphosate molecule and for the slab system with the anionic form of glyphosate.

**TABLE 1:** Adsorption free energy of glyphosate species on the aluminol surface of kaolinite

System	Δ*G* ^0^ kJ mol^−1^
C_3_H_8_NO_5_P (periodic)	−5.0 ± 0.1
C_3_H_8_NO_5_P (slab)	−5.1 ± 0.1
C_3_H_7_NO_5_P^−^ (slab)	−14.5 ± 0.2

As expected, anionic glyphosate is adsorbed more strongly than neutral glyphosate, with an adsorption free energy about three times larger than in the models with the neutral glyphosate molecule. On the other hand, the difference in the adsorption free energy for the neutral molecule in the periodic and slab models is negligible (0.1 kJ mol^−1^). The strong attraction of the glyphosate anion to the aluminol surface was confirmed by letting the glyphosate ion in the polarized periodic model move freely during the MD simulation, starting with the ion in the middle of the simulation box. Shortly after the simulation started the glyphosate ion diffused through the water slab and attached to the aluminol surface. In contrast, both the neutral and ionic states of glyphosate in the slab models did not experience any strong driving force towards any of the surfaces.

## 4 DISCUSSION

Previously, we demonstrated that ionic solutions polarized the system (Galicia‐Andrés et al., 2019). Considering that adsorption of ionic glyphosate is enhanced in polarized systems, we can hypothesize that saturated surfaces will enhance even more adsorption of glyphosate on mineral clays, similar to the experimental results reported by McConnell and Hossner (1985) and Glass (1987).

**FIGURE 5:**
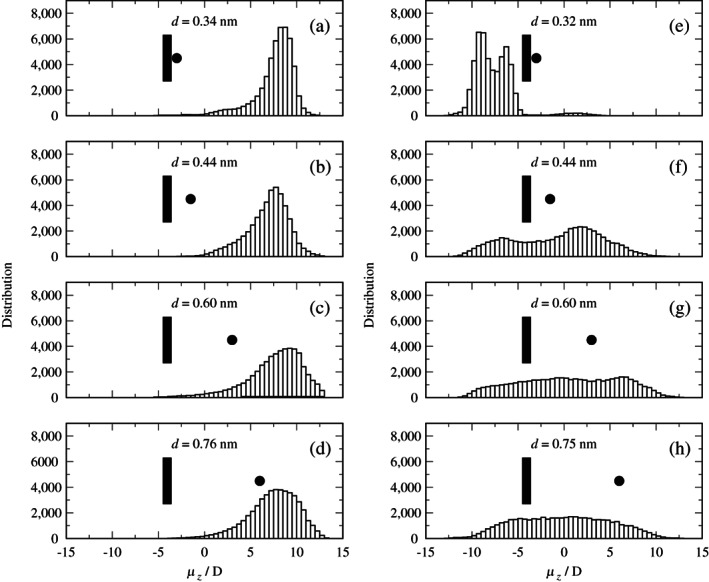
Probability distribution of *z*‐component of the dipole moment of neutral glyphosate at distances from the kaolinite layer of the periodic (left panels) and slab (right panels) models

To analyse in more detail the role of polarization in glyphosate conformations in adsorption states, we exhibit in Figure 5 (right panels) the projection of the dipole moment of the neutral glyphosate molecule on the *z*‐axis from the umbrella sampling trajectories corresponding to the observed minima of the PMF curves of the slab model (Figure 4) and a point located just before reaching the plateau start (0.71 nm). In addition, the left panels in Figure 5 correspond to the dipole moment distributions for the periodic model at the same distances. It is evident from the well‐localized peaks that in the case of the periodic model there is a preferential orientation of the glyphosate molecule with respect to the kaolinite surface; the distributions only become broader as the molecule moves further from the surface. Observed maxima are in a range of 7.5–9.0 D, pointing to an alignment of the molecular dipole moment in the *z*‐direction, along the induced electric field.

For the slab model, a completely different behaviour is observed (right panels in Figure 5). A bimodal curve (Figure 5e) with a small shoulder appeared for glyphosate localized in the global minimum. However, the observed negative values of the dipole moment, in contrast to the periodic model, (Figure 5a), indicate that new conformations were accessible due to the absence of polarization. As the distance between glyphosate and kaolinite increased, the intensity of both maxima decreased, with a strong broadening of the interval of the distributions and centring them at about 0 D (Figure 5f–h), confirming that the glyphosate molecule is freely tumbling in the slab model. However, the alignment of the glyphosate dipole moment in periodic models, due to the polarization effect, restricts conformational degrees of freedom for polar molecules, explaining why observed minima on the periodic and slab PMFs do not perfectly match (Figure 4). For the charged species, very similar observations were made (see Figure S1 in Supplementary Information).

The polarization effects involve a collective effect on all species present in the system, and on the molecular conformation of glyphosate in the adsorption mechanism. To discriminate the contribution of directional short‐ranged interactions (i.e. H‐bonds), which are precursors of a surface complex formation, to the interaction energy between glyphosate and the kaolinite aluminol surface, we performed an analysis of the distribution of the non‐bonding interaction energy and H‐bonding with respect to individual functional groups of glyphosate at selected distances between the surface and the COM of the glyphosate molecule. Distances were selected based on the features of the PMFs.

**FIGURE 6:**
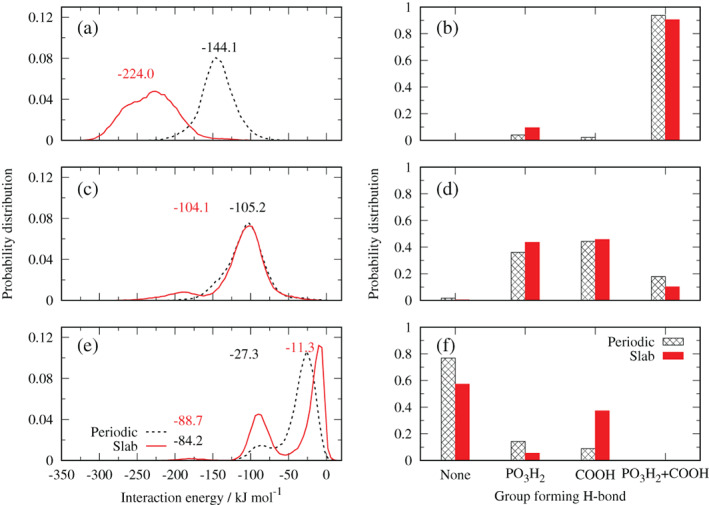
Interaction energy distribution of kaolinite and neutral glyphosate (left panels) and H‐bond distribution of polar groups in glyphosate (right panels) at different average distances: (a) and (b) 0.33 nm; (c) and (d) 0.44 nm; (e) and (f) 0.60 nm [Color figure can be viewed at wileyonlinelibrary.com]

The results are collected in Figure 6. Panels on the left side represent the non‐bonded interaction energy histograms, with a normal distribution behaviour. The right side of Figure 6 shows the occurrence of H‐bonds formed between the particular groups of neutral glyphosate (carboxylic and phosphonic). The amino group is a proton donor and its H‐bond capacity depends on aluminol O atoms being in a favourable orientation to act as proton acceptors. When the glyphosate molecule is at an average distance of 0.33 nm from the aluminol surface, a maximum in the interaction energy distribution is observed at −144 kJ mol^−1^ for the periodic model, whereas for the slab model the maximum is located at −224 kJ mol^−1^ (Figure 6a). These values are similar to the outer‐sphere complex interaction energy, ~ −180 kJ mol^−1^, reported by Ahmed et al. for the glyphosate‐(100) goethite surface plane (Ahmed et al., 2018). Because both glyphosate functional groups are bonded simultaneously most of the time (Figure 6b), it can be inferred that the glyphosate molecule is oriented mostly parallel to the kaolinite surface, as shown in Figure 3b. Then, the observed difference in energy is mainly attributed to glyphosate conformational degrees of freedom, which do not exclude polarization effects, H‐bonds and interactions with surrounding water molecules.

With the increasing distance between the COM of glyphosate and the kaolinite surface, the interaction energy and the probability of H‐bonding decreases. In Figure 6c it is observed that both systems have a similar distribution at a distance of 0.44 nm, with a maximum at ~ −104 kJ mol^−1^. In this configuration both functional groups contribute to the H‐bonding more or less equally (Figure 6d). This is also reflected in a close distance between the PMF curves at a distance of 0.44 nm in Figure 4. We suppose that the glyphosate molecule has a more or less perpendicular orientation with respect to the surface, switching its binding to the surface once linked either with the phosphonate or the carboxylate group in both the periodic and slab models, and this orientation is mostly responsible for the energetic interactions, without an apparent contribution from the dipole moment, in contrast to the previous situation.

At a distance of ~0.6 nm both models exhibit a bimodal behaviour (Figure 6e). The global maximum is located at −27.3 and −11.3 kJ mol^−1^ for the periodic and slab models, respectively, where none of the functional groups H‐bonds with the surface (Figure 6f). As discussed previously, energy differences are mainly attributed to the glyphosate conformation, where the polarization effect influences orientation of the molecular dipole moment (Figure 5c,g). For the second maximum, a small shoulder in the periodic model is centred at −84.2 kJ mol^−1^, which corresponds to low probability of glyphosate H‐bonding with kaolinite; similarly, close to that energy value, the slab model has a better resolved peak at −88.7 kJ mol^−1^ that is attributed to the COOH group. However, the difference can be attributed to the number of sampled conformations that are limited for the polarized situation in the periodic system.

**FIGURE 7:**
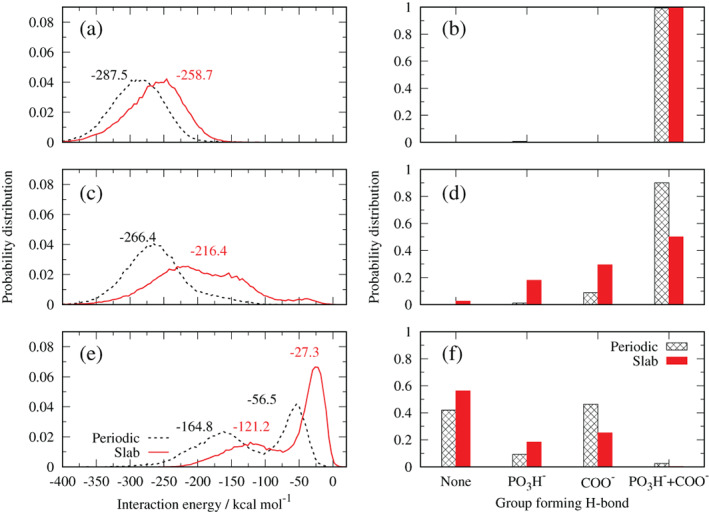
Interaction energy distribution of kaolinite and glyphosate anion (left panels) and H‐bond distribution of polar groups in glyphosate (right panels) at different average distances: (a) and (b) 0.33 nm; (c) and d) 0.37 nm; (e) and (f) 0.55 nm [Color figure can be viewed at wileyonlinelibrary.com]

Figure 7 shows similar plots for the glyphosate ion to those shown in Figure 6 for the neutral molecule. We observed similar energy distributions for periodic and slab models at the closest distance, with lower values (−287.5 and −258.7 kJ mol^−1^) compared to the neutral molecule, pointing to a stronger attraction with the surface OH groups. In this configuration, both phosphonate and carboxylate groups contribute equally to the H‐bonding (Figure 7b). Still, the effect of the glyphosate conformations must be present. However, because electrostatic interactions are stronger due to the ionic nature of glyphosate, they dominate, making the energy difference smaller compared to neutral glyphosate at the same distance (Figure 6a). Comparing energy distributions at distances of 0.33 and 0.37 nm (Figures 7a‐c), a shift of ~20 kJ mol^−1^ is observed in the periodic model, whereas for the slab model, the maximum is shifted by ~40 kJ mol^−1^. A new shoulder of the energy distribution is observed in both systems, being more evident for the slab model. This shoulder is attributed to the H‐bonding of any of the functional groups, being more evident for the slab model (Figure 7d). We suppose that electrostatic interactions still dominate over other effects in the periodic model, whereas in the slab model conformational effects start to be dominant, which is spotted as a second minimum at 0.44 nm of the PMF curve (Figure 4b). Finally, in Figure 7e a similar behaviour to the neutral glyphosate is observed, with distinguishable maxima at −56.5 and −164.8 kJ mol^−1^ (periodic model), and −27.4 and −121.2 kJ mol^−1^ (slab model), respectively. Considering that the COO^−^ group is mostly responsible for the glyphosate net charge it is expected that it interacts more strongly with the surface OH groups than the phosphonate group (Figure 7f). Moreover, when polarization is present (periodic model), H‐bonds of the COO^−^ group occur most of the time, with more than 40% of probability (for the slab model it is more than 20%). We hypothesize that the observed shift between both models is caused by the long‐range interactions from particular conformations adopted by the anionic glyphosate, induced by the polarization effects. This affinity to kaolinite supports experimental work carried out in volcanic ash soils that show the strong adsorption of glyphosate to kaolinite (Cáceres‐Jensen, Gan, Báez, Fuentes, & Escudey, 2009).

Extrapolating our results, we hypothesize that organic matter, characterized as supramolecular agglomerates with an aliphatic backbone, aromatic moieties and functional polar groups (Aquino et al., 2011) in contact with other small molecules (Petrov, Tunega, Gerzabek, 2017; Petrov, Tunega, Gerzabek, & Oostenbrink, 2019; Sündermann, Solc, Tunega, Haberhauer, Gerzabek, 2015) behaves like the glyphosate. This behaviour can explain the strong adsorption and stabilization mechanisms of organic matter on clay minerals (Six, Conant, Paul, & Paustian, 2002; Vogel et al., 2015).

## 5 CONCLUSION

In summary, different aspects were identified as responsible for the glyphosate adsorption process from water solution to the aluminol kaolinite surface, as well as its enhancement; among them were: (a) polarization effects, which involve collective effects of all present molecules; (b) molecular conformation of the adsorbed species; (c) directional short‐ranged interactions such as H‐bonds; and (d) net charge of the adsorbed moiety.

We demonstrated that adsorption free energy of the neutral species is only minimally affected by polarization effects. However, it influences the alignment and conformational degrees of freedom of the molecule due to long‐range electrostatic interactions (charges and dipole moments), enhancing the adsorption processes of the ionic glyphosate form. In general, it was shown that the strength of adsorption of glyphosate to the kaolinite surface is strongly pH dependent. Adsorption energy and non‐bonded interaction energy are higher for anionic glyphosate than neutral glyphosate, suggesting that at higher pH values, the glyphosate moiety can be strongly adsorbed. On the other hand, at low pH values, glyphosate is neutral and its adsorption is weaker, inducing a higher mobility of the molecule.

We observed that these interactions are responsible for long‐lasting H‐bonds and how they are formed. We identified that close to kaolinite, the glyphosate molecule has a parallel orientation with respect to the surface and H‐bonding of all polar functional groups, with the surface OH groups contributing to the relatively strong surface complexation. H‐bond formation is affected by polarization effects, being more evident for the anionic form of glyphosate. Using molecular simulations, we identified the contributions of two glyphosate species (neutral and −1 charged) to the adsorption process with the aluminol surface of the kaolinite clay mineral in presence of water at molecular scale. Our study revealed that glyphosate, specifically in its anionic form, binds with a significant affinity to kaolinite, a major soil mineral constituent in weathered tropical and subtropical soils, supporting experimental results.

## CONFLICT OF INTEREST

All authors declare that there is no conflict of interest.

## AUTHOR CONTRIBUTIONS

Study concept and design: all. Acquisition of data: E.G.‐A. and D.T. Analysis and interpretation of data: E.G.‐A., D.T. and C.O. Drafting the manuscript: E.G.‐A. Critical revision of the final manuscript: all.

## Supporting information


**Appendix S1**. Supporting Information.

## Data Availability

All relevant data is available from the authors upon request.
