# Supplementary material for: On glyphosate–kaolinite surface interactions. A molecular dynamic study
Source: Eur J Soil Sci. 2020 May 6;72(3):1231–42. doi: 10.1111/ejss.12971 (PMC8247318; doi:10.1111/ejss.12971)
Supplement: Supplementary file 1 — Appendix S1. Supporting Information. [file EJSS-72-1231-s001.pdf]

Supporting information for: On glyphosate–kaolinite surface interactions. A molecular dynamic study

Edgar Galicia-Andrés,[*,†,‡] Daniel Tunega,[*,‡,¶] Martin H. Gerzabek,[‡] and Chris Oostenbrink[*,†]

†*Institute of Molecular Modeling and Simulation, University of Natural Resources and Life Sciences, Muthgasse 18, 1190 Vienna, Austria*

‡*Institute of Soil Research, University of Natural Resources and Life Sciences, Peter-Jordan-Straße 82, 1190 Viena, Austria*

¶*School of Pharmaceutical Science and Technology, Tianjin University, 92 Weijin Road, Nankai District, Tianjin, P.R. China*

E-mail: edgar.galicia@boku.ac.at; daniel.tunega@boku.ac.at; chris.oostenbrink@boku.ac.at

Phone: +43 1 47654-89417; +43 1 47654-91148; +43 1 47654-89411

Standard adsorption free energy

We calculated the standard adsorption free energy following a procedure similar to that described by Doudou et al.[1] The adsorption free energy is equivalent to a reaction free energy of the following chemical reaction:

$$\mathrm{K_{(s)}} + \mathrm{G_{(l)}} \rightleftharpoons \mathrm{KG_{(s)}} \tag{1}$$

with $\mathrm{K_{(s)}}$ and $\mathrm{KG_{(s)}}$ being kaolinite and kaolinite–glyphosate species as a solid phase, and $\mathrm{G_{(l)}}$ being glyphosate molecule in water solution. By using the definition of the chemical potential of i, μ_i, in terms of the activity a_i,

$$\mu_i = \mu_i^0 + k_B T \ln a_i, \tag{2}$$

where k_B is the Boltzmann constant and T is the temperature, it is possible to write ΔG as

$$\begin{aligned} \Delta G &= \mu_{\mathrm{KG}} - \mu_{\mathrm{K}} - \mu_{\mathrm{G}} \\ &= \Delta G^0 + k_B T \ln \frac{a_{\mathrm{KG}}}{a_{\mathrm{K}} a_{\mathrm{G}}} \end{aligned} \tag{3}$$

where the superscript 0 states for the standard state and the activity can be expressed in terms of molar concentration of the i species (C_i),

$$a_i = \gamma_i \frac{C_i}{C^0}, \tag{4}$$

where C^0 is a reference molar concentration and γ_i the activity coefficient. For dilute systems the activity coefficient of solute i is

$$\lim_{C_i \to 0} \gamma_i = 1 \tag{5}$$

For the solid phases we can write

$$C_{\mathrm{KG}} \approx C_{\mathrm{K}} = \frac{1}{V}, \tag{6}$$

and

$$C_{\mathrm{G}} = \frac{1}{V_{\mathrm{unbound}}}, \tag{7}$$

with V being the volume of the simulation box and V_{unbound} the molar volume of the free glyphosate in a water slab, i.e., in the unbound state, and

$$C^0 = \frac{1}{V^0}, \tag{8}$$

where the standard molar volume is $V^0 = 1.661$ nm^3. The adsorption free energy can be expressed as

$$\begin{aligned}\Delta G &= \Delta G^0 + k_B T \ln \frac{V_{\mathrm{unbound}}}{V^0} \\ &= -k_B T \ln \int_{z \in \mathrm{bound}} \exp\left[-\frac{\Delta G_{\mathrm{PMF}}(z)}{k_B T}\right] dz + k_B T \ln \int_{z \in \mathrm{unbound}} \exp\left[-\frac{\Delta G_{\mathrm{PMF}}(z)}{k_B T}\right] dz\end{aligned} \tag{9}$$

where ΔG_{PMF} is the PMFs contribution. The integral terms are solved for the bound and unbound regions, where the free energy corresponding to the standard volume change is defined as

$$\Delta G_{\mathrm{V}} = -k_B T \ln \frac{V_{\mathrm{unbound}}}{V^0}. \tag{10}$$

Finally the standard adsorption free energy may be written as

$$\Delta G^0 = \Delta G + \Delta G_{\mathrm{V}}. \tag{11}$$

Supplementary figures

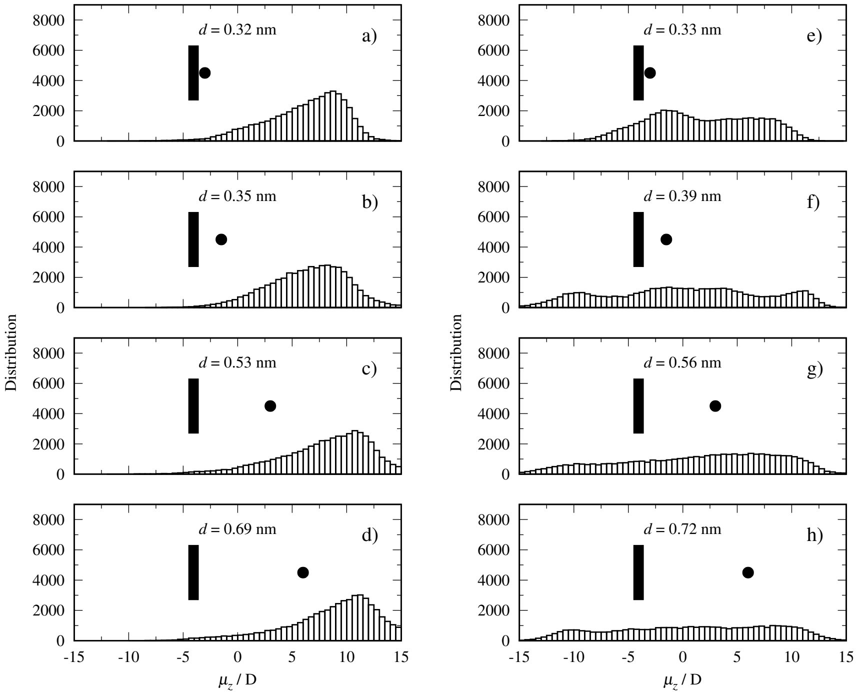

Figure S 1: Probability distribution of z–component of the dipole moment of charged glyphosate at distances from the kaolinite layer of the periodic (left panels) and slab (right panels) models.

References

(1) Doudou, S.; Burton, N. A.; Henchman, R. H. Standard Free Energy of Binding from a One-Dimensional Potential of Mean Force. *Journal of Chemical Theory and Computation* **2009**, *5*, 909–918, PMID: 26609600.
